# Cost-effectiveness analysis of a strategy to delay progression to dialysis and death among chronic kidney disease patients in Lima, Peru

**DOI:** 10.1186/s12962-021-00317-0

**Published:** 2021-10-10

**Authors:** E. M. Saldarriaga, J. Bravo-Zúñiga, Y. Hurtado-Roca, V. Suarez

**Affiliations:** 1 Instituto de Evaluación de Tecnologías en Salud E Investigación (IETSI), EsSalud, Av. Arenales 1302, office 310, Lima, Perú; 2 The Comparative Health Outcomes, Policy and Economics (CHOICE) Institute, University of Washington, Seattle, USA; 3 Sin Brechas S.A.C., Lima, Perú; 4 Departamento de Nefrología, Unidad de Salud Renal, Hospital Nacional Edgardo Rebagliati Martins, EsSalud, Lima, Perú

**Keywords:** Prevention of chronic kidney disease, Cost-effectiveness analysis, CKD progression delay

## Abstract

**Background:**

The Renal Health Program (RHP) was implemented in 2013 as a secondary prevention strategy to reduce the incidence of patients initiating dialysis and overall mortality. A previous study found that adherent patients have 58% protection against progression to dialysis compared to non-adherent. The main objective of the study was to estimate the lifetime economic and health consequences of the RHP intervention to determine its cost-effectiveness in comparison with usual care.

**Methods:**

We use a Markov model of three health stages to simulate disease progression among chronic kidney disease patients in Lima, Peru. The simulation time-horizon was 30 years to capture the lifetime cost and health consequences comparing the RHP to usual care. Costs were estimated from the payer perspective using institutional data. Health outcomes included years lived free of dialysis (YL) and quality adjusted life years (QALY). We conducted a probabilistic sensitivity analysis (PSA) to assess the robustness of our estimates against parameter uncertainty.

**Results:**

We found that the RHP was dominant—cost-saving and more effective—compared to usual care. The RHP was 783USD cheaper than the standard of care and created 0.04 additional QALYs, per person. The Incremental Cost-Effectiveness Ratio (ICER) showed a cost per QALY gained of $21,660USD. In the PSA the RHP was dominant in 996 out of 1000 evaluated scenarios.

**Conclusions:**

The RHP was cheaper than the standard of care and more effective due to a reduction in the incidence of patients progressing to dialysis, which is a very expensive treatment and many times inaccessible. We aim these results to help in the decision-making process of scaling-up and investment of similar strategies in Peru. Our results help to increase the evidence in Latin America where there is a lack of information in the long-term consequences of clinical-management-based prevention strategies for CKD patients.

**Supplementary Information:**

The online version contains supplementary material available at 10.1186/s12962-021-00317-0.

## Introduction

The prevalence of chronic kidney disease (CKD) in Lima, capital of Peru, is 21% [1]; 7% higher than the national mean [2]. Each year, 51 thousand life years are lost due to CKD and associated complications [3]. Patients that progress to the end stage of the renal disease (ESRD) are likely to need renal replacement therapy (RRT). In Peru, the most prevalent RRT option is hemodialysis [2], whose annual cost ranges from $3424 to $42,785 [4]. This cost can be prohibitive for many patients. It has been reported that only 50% of Peruvians in need of hemodialysis are able to obtain treatment [5]. The elevated cost of RRT not only causes healthcare access barriers, but it also carries major financial burden for healthcare providers [6].

Embedded in this context, the Renal Health Unit of the Hospital E. Rebagliati—the biggest facility of the Peruvian social health insurance (EsSalud)—implemented a secondary prevention intervention in 2013 called the Renal Health Program (RHP) [7]. The RHP aimed to reduce the incidence of patients progressing to dialysis and overall mortality. The intervention focused on systematic control of estimated Glomerular Filtration Rate (eGFR) and microalbuminuria for all CKD patients, comprehensive healthcare attention to control for comorbidities, and promotion of healthy lifestyles. This holistic approximation to the CKD requires coordinated efforts between the primary and the specialized care and hence aligns with the evidence that this approach is an effective way to detect at-risk patients [7].

The efficacy of the RHP was evaluated in competitive-risk survival analysis [8]. This study showed an effectiveness of 58% in reducing the risk of progression to dialysis. There was no significant effect in the overall mortality. The current analysis builds on the said epidemiological results to inform a cost-effectiveness analysis (CEA). Our study aims to provide an economic value assessment of the RHP intervention from the payer perspective.

The economic evaluation is an essential piece to inform the decision-making process in Peru. In addition, our findings can be useful to other countries in Latin America, a region with limited coverage of CKD treatment and dialysis for which this type of intervention might be attractive [9, 10].

## Methods

### Intervention

The RHP intervention is based on case-management of CKD patients through the frequent and regular observance of the eGFR and microalbuminuria levels to prevent the progression of the renal disease, control of comorbidities status (diabetes and hypertension), and promotion of healthy lifestyles (better nutrition and exercise habits) [7]. The RHP defined the frequency of office appointments, testing, and rules for referral to specialized care. Conversely, patients in the standard of care did not have frequent follow up. They obtained outpatient visits on-demand and subject to availability. Additional details about the characteristics of care in the intervention have been provided in previous published studies [7], as well as the main differences between the intervention and control groups [8].

### Setting

The RHP was implemented in the Hospital E. Rebagliati Network. This includes the national Hospital E. Rebagliati, that receives patients from all over the country once their case is complex enough and warrants a referral. In addition, it includes primary care facilities with local reach [11].

### Target population

Target patients were adults 18 years old or above, with a diagnosis of CKD. Patients were classified in 5 states of disease progression according to the KDIGO guidelines [12] which determines specific aspects of the intervention delivery. In each visit, they received attention from the physician, nurse, and nutritionist, and performed an eGFR test for microalbuminuria detection. Additionally, some patients received social assistant and psychology care. Patients were required to visit a primary care facility in a variant frequency depending on their CKD stage: once a year for stages 1 and 2, twice for stages 3a and b, and three times for patients in stage 4. Patients in the early stages of the CKD (1–3a) must receive attention in primary care, while late-stage patients receive attention in specialized facilities. The analysis was performed for the entire cohort, no sub-groups were defined.

### Modelling approach

**Fig. 1:**
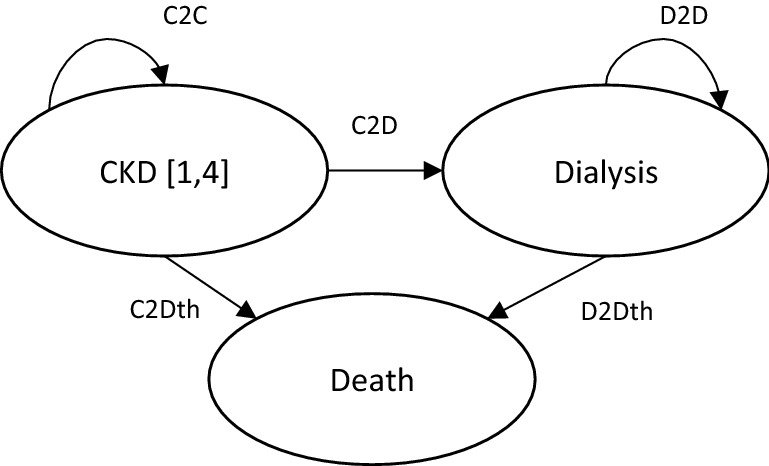
Markov model and transition probabilities codes. *CKD* chronic kidney disease. Includes stages 1–4 of the disease

We performed a deterministic CEA comparing the cost and health consequences of the RHP to the standard of care. We used a Markov model defined by three health states: CKD (all stages), dialysis and death (Fig. 1). This is a compartmental model where the cohort transits across the health states at rates defined by transition probabilities; the probability of moving across states is given only by the current state, and hence no historic information is used [13].

For consistency with the time-to-event outcomes, the transition probabilities in the Markov model were estimated from fitted survival curves and informed by the previous epidemiological study that assessed the effectiveness of the RHP. We found two parametric survival curves to define the transitions from the “CKD” compartment to “Dialysis” and “Death” in the control group. For each survival curve, we identified a set of potential distributions including Weibull, exponential, logistic, log-normal, and log-logistic. The best fit to the observed data was the distribution with the lowest Akaike and Bayesian Information Criteria (AIC and BIC, respectively). In addition, we used a visual representation of the data to assess how well the curves fi the observed data.

Using the results of the selected distribution we estimate the lambda and gamma parameters as: $1/\left[ {\exp \left( {intercept} \right) \wedge \left( {1/scale} \right)} \right]$ and $1/scale$, respectively. These two parameters allowed us to estimate the survival probabilities from CKD to dialysis and death for each cycle in the usual care following this formula: $1 - \exp \left[ {lambda \times \left( {cycle - 1} \right)^{gamma} - cycle^{gamma} } \right].$ Thus, this is the probability of transition from the first health state to the other ones, expressed as a function of the cycle that accounts for the increasing risk to event over time. The time scale in the previous study was days, in this study we used years for convenience given the time-horizon of the analysis. We fitted survival curves under both scales obtaining very similar results. We found that the Weibull distribution had the best fit among the evaluated parametric curves for both progressions to dialysis and mortality. For event-free survival the estimated lambda and gamma were 0.008 and 0.938, respectively; and 0.043 and 1.143, for the mortality curve. The numerical and graphical results of the calibration process for both curves are display in the Additional file 1.

For the intervention, we adjusted the survival curves by the estimated treatment effects; the hazard ratios found in the epidemiological study: 58% (HR  =  0.42, 95% CI 0.21, 0.71) reduction in risk to progress to dialysis and no change in mortality risk (HR  =  1, 95% CI 0.88, 1.13) [8]. Given that the target population of the epidemiological study and the CEA are the same, these treatment effect estimates have enough internal validity to create reliable results. However, we given the inherit uncertainty of the estimates, we will include both treatment effects in the sensitivity analysis.

The probability of transition from dialysis to death was estimated through literature review. We found two studies that estimated mortality rates after RTT initiation. One reported a survival probability of 95% for the first year of dialysis, 91% for the second, and 88% for the third [14]. Since our model only considers people that just started in dialysis, and the model has a memoryless property, we use a fixed rate of 5% to estimate this transition.

Considering that the mean age of the population of interest is around 60 years old [8], our model ran for 30 cycles, where each cycle represents one year, to accrue the lifetime health and economic consequences of the intervention. According to country-specific life tables, after 90 years of age, less than 10% of the Peruvian population would still be alive [15]. Cost and outcomes were observed at the end of each cycle. We used a simulated cohort of 1000 people for each strategy, replicating the same CKD stage distribution and diabetes prevalence as in the original dataset for representative purposes and internal validity.

### Cost assessment

Costs were estimated from the payer perspective, EsSalud, considering direct medical costs for both alternatives. We considered all costs faced by the payer to provide treatment in one year. For the intervention, we considered the costs of nephroprotection treatment, outpatient visits, and laboratory tests. These costs are not homogeneous across stages of CKD but vary due to frequency of provision (hospital visits and lab tests), and type of facility (primary care facilities have cheaper provision costs than more specialized ones), and patients with diabetes receive additionally glycosylated hemoglobin tests. We also included the implementation cost of the intervention, including a first investment that includes the time utilized by the Renal Health Unit to develop the intervention, protocol, guidelines, personnel training, and a yearly operational cost that includes the time spent by the RHP team identifying, testing, referring, and keeping accurate records of the patients.

While the patients undergoing intervention followed a treatment protocol and were closely followed-up, patients on the standard of care receive on-demand outpatient visits. Testing was subject to medical indication. There was not a fixed frequency for neither of those services, contrary to the intervention. Thus, healthcare utilization would depend on the patients’ behavior and availability. Given the inherent randomness of this healthcare utilization variables, we decided to base our estimates of frequency of care reception on expert consultation. The nephrologists from the Renal Health Unit provided us with their best-educated guess of the number of outpatient visits that a regular CKD patient receives in one year. We also added the cost of one laboratory test per year without differentiation for diabetes condition or facility in which it would take place. This allowed us to keep the usual care costs low, to obtain conservative estimations for the incremental cost-effectiveness ratio (ICER).

The treatment costs varied across CKD stages in both alternatives, and across diabetes status in the RHP. Given that the Markov model included one compartment for all CKD stages, we used a weighted average to estimate the annual treatment cost per patient in each alternative. The weight was defined by the proportion of stages and diabetic patients in the observed data. Finally, we included the annual cost of hemodialysis as the product of the cost per session plus the drugs prescribed in each one and the number of sessions in a year.

We used two sources of costing data: the EsSalud General Management Office cost report (2018), and the report of resources use specifically for the intervention from the Renal Health Unit (2014). The first one provided the unit cost per activity, while the latter gives us the number of units per activity consumed to follow and treat a regular patient in each CKD stage. We used institutional costs to reduce uncertainty around the final estimations. Data collection was conducted in local currency, Peruvian Soles (PEN), while results are presented in United States Dollars (USD, $).

### Health consequences

We sought to compare the differences in health outcomes between the alternatives using years lived free of dialysis (YL) and Quality Adjusted Life Years (QALY), to obtain a measure of the number of person-years avoided in dialysis and the number of person-years of perfect health gained, associated with the adherence to the intervention. Form literature review we defined the utility score for CKD patients in 0.84, and for patients starting dialysis in 0.65 [16]. In the study, these scores correspond to the stage 3, the most prevalent stage in our cohort, and to the stage 5, a close approximation to patients just starting RRT.

### Analysis

We projected the costs and health outcomes of each alternative separately during 30 cycles, to capture life-time consequences. Cost and health outcomes would be discounted by an annual rate of 3% to reflect the time-preferences of the economic agents. After these calculations, we aggregated the total costs, YL and QALY from each alternative and express them in per-person units.

To determine which alternative poses the highest economic value we used the ICER calculated as the difference in cost between RHP and usual care over the differences in health outcomes. Then, we can interpret the ICER as the additional cost for the payer to avoid one person-year in dialysis and to gain one QALY. Cost estimations were made in local currency but converted to USD using a fixed exchange rate of 3.3 PEN per each USD, corresponding to the annual average for 2018 [17]. We used the cost-effectiveness threshold of 1–3 times the gross domestic product (GDP) per capita, estimated in $6571 for Peru, according to the World Bank [18].

To assess the robustness of the ICER against parameter uncertainty we performed a Probabilistic Sensitivity Analysis (PSA) based on a Monte Carlo simulation of 1000 repetitions. In each repetition, the model randomly picked a value for each varying parameter considering its distribution and range of values. Each repletion represents a unique scenario for the comparison of RHP and the standard of care. Table 1 shows a summary of the parameters used in the study and the values they took in the sensitivity analysis. The range of values for each parameter was as follows: the treatment effects would take the lower and upper values of the estimated confidence interval, the costs would vary 15%, the utilities would change 10%, and the discount rate would take 0% to reflect no discounting and 5% to reflect a scenario with higher opportunity cost. We summarized the results by descriptive statistics of the incremental cost per QALY and a figure showing the incremental costs and QALYs for each simulation. We report central tendency values for the distribution of both the incremental cost and effectiveness, including mean, standard deviation (SD), relative standard error $\left( {RSE = SD/mean \times 100} \right)$ expressed in percentages, and range (min. and max.) of values.

**Table 1:** Study parameters

Survival projection	Value	Sensitivity range	Source
Dialysis (probability C2D)			
Treatment effect (hazard ratio)	0.42	0.21–0.71	Bravo-Zúñiga et al. [8]
Lambda^a^	0.008	0.007–0.009	Estimated
Gamma^a^	0.938	0.925–0.952	Estimated
Mortality (probability C2Dth)			
Treatment effect (hazard ratio)	1	0.88–1.13	Bravo-Zúñiga et al. [8]
Lambda^a^	0.043	0.034–0.053	Estimated
Gamma^a^	1.143	1.098–1.193	Estimated
Mortality among dialysis patients (probability D2Dth)	0.05		Cieza-Zevallos et al. [14]
Annual costs (USD)^b^			Estimated (see Table 2)
RHP cost of treatment	531	452–611	
Standard of care cost of treatment	45	38–52	
Dialysis treatment	13,459	11,440–15,478	
RHP Initial investment (one-time)	30,412		
RHP annual operational costs	4995		
Utility scores			Go et al. [16]
CKD event-free	0.84	0.76–0.92	
First time dialysis	0.65	0.59–0.72	
General			
Discount rate costs and outcomes (%)	3	0–5	
Exchange rate (PEN per USD)	3.3		Central Reserve Bank of Peru [17]
Projection length (cycles)	30		
Cycle length (years)	1		

*RHP* Renal Health Program; *CKD* chronic kidney disease; *USD* United States Dollars; *PEN* Peruvian Soles

^a^Values rounded to the third decimal

^b^All costing data was rounded to the closest integer

The analysis and assessment of parametric distributions to fit the data was performed in R studio, the CEA and PSA were developed in TreeAge^®^ [19].

## Results

**Fig. 2:**
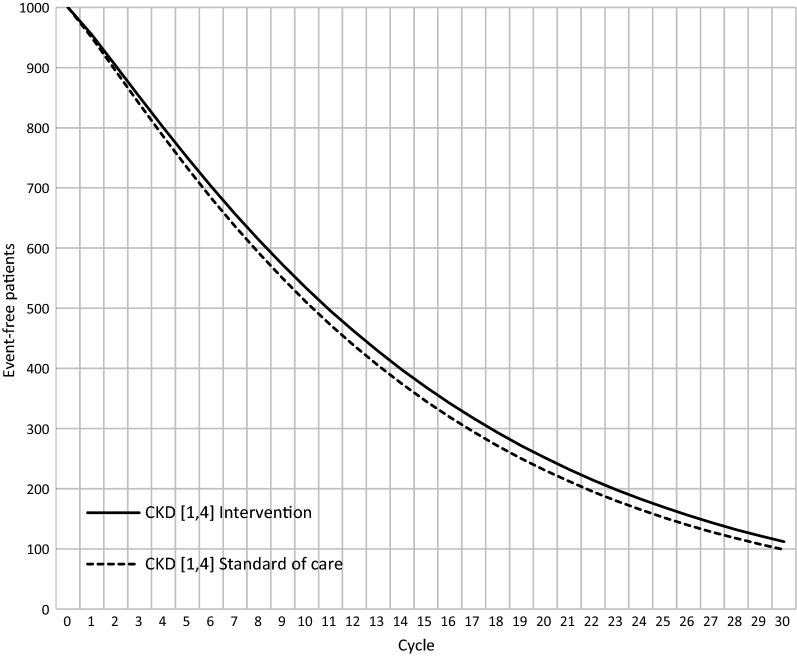
Event-free survival by alternative. *CKD* Chronic Kidney disease. Includes stages 1–4 of the disease

We found that the event-free survival curve of the intervened cohort is constantly and slightly over the usual care (Fig. 2). Indicating that the intervention produced gains in years lived without dialysis and QALYs.

Based on the institutional costing data, we estimate the annual per-patient cost of treatment is $531.18 for the RHP alternative, and $45.18 in usual care (Table 2). The annual cost of dialysis is $13,458.79 per patient, composed by 156 sessions at $79.09 each plus $6.8 for drugs in each session, and 4 medical appointments in Nephrology ($14.84 each). The initial implementation cost of RHP is $30,411.52 and the annual operation cost $4994.8 (Table 1). We do not express the operational cost as per person because they are mostly composed of fixed costs with negligible marginal costs per patient. Thus, in each alternative, the costs of the provision in one year would be determined by the proportion of people remaining event-free multiplied by the cost of treatment, plus the proportion of people entering dialysis, multiplied by the cost of one year of therapy. Additionally, for the RHP we add the cost operational cost.

**Table 2:** Treatment cost for each stage and alternative

	Stage 1		Stage 2		Stage 3a		Stage 3b		Stage 4	
	Diabetic patient (D)	Non-diabetic patient (ND)	D	ND	D	ND	D	ND	D	ND
Cohort distribution										
Proportion of diabetic patients within stage (%)	51	49	42	58	29	71	24	76	22	78
Number of diabetic patients within stage (n)	1833	1781	2109	2890	2407	6026	536	1671	245	856
Proportion of patients in each stage^a^ (%)	18		25		41		11		5	
Treatment cost intervention (USD)^b^										
Outpatient visits	315	315	315	315	340	340	439	370	1018	840
Laboratory tests	38	34	38	34	69	62	270	197	344	297
Drugs	47	43	47	43	47	43	118	84	335	280
Hospital overhead	20	20	20	20	20	20	40	40	40	40
Total	420	412	420	412	476	464	867	691	1736	1456
Annual cost per patient for each stage^a^	416		415		468		734		1519	
Annual cost per patient^c^	531									
Treatment cost standard of care (USD)^b^										
Outpatient visits	15		15		15		30		30	
Laboratory test	28		28		28		28		28	
Total	43		43		43		58		58	
Annual cost per patient^c^	45									

^a^Value corresponds to each stage, groups across diabetes status

^b^All costing data was rounded to the closest integer

^c^Value corresponds to all stages and diabetes status

Our results showed that after 30-year simulation, the RHP was $783 per person cheaper than the standard of care. The RHP created an additional 0.36 event-free years and 0.04 QALY per person. We found a cost per QALY of $21,660, and $2173 per year avoided in dialysis. The RHP is a dominant alternative with lower provision costs than the standard of care and higher health outcomes (Table 3).

**Table 3:** Cost-effectiveness analysis results

	Renal Health Program	Standard of care	Difference
Costs (USD, per person)	9357	10,140	− 783
YL (per person)	9.50	9.14	0.36
QALY (per person)	8.19	8.15	0.04
ICER: USD/YL	− 2173		
ICER: USD/QALY	− 21,660		

*QALY* quality-adjusted life year; *ICER* incremental cost-effectiveness ratio; *YL* years of life; *USD* United States Dollars

Costs and ICER values were rounded to the closest integer; YL and QALY values were rounded to two decimals

### Sensitivity analysis

**Fig. 3:**
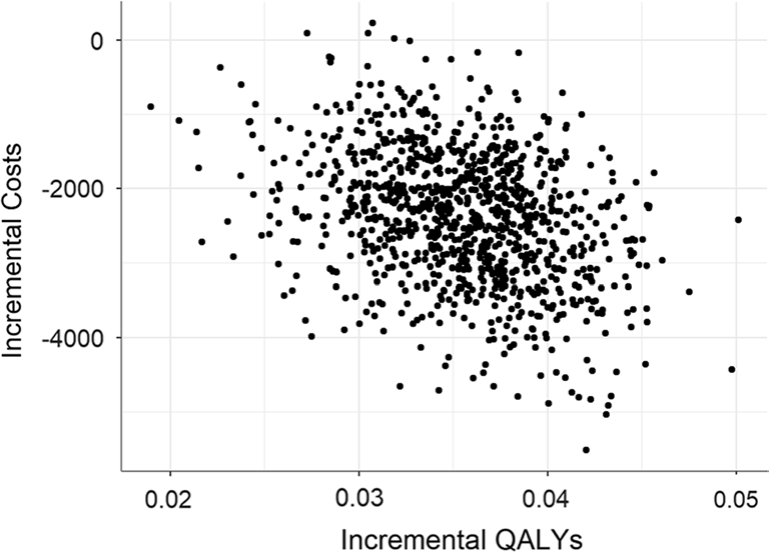
Probabilistic sensitivity analysis. The x-axis expresses the incremental quality adjusted life-years (QALYs) comparing the Renal Health Program (RHP) to standard of care, while the y-axis represents the incremental costs of the same comparison

From the PSA we found that standard of care showed consistently higher costs and similar QALYs than the RHP. The mean cost per QALY was $20,309, with a SD of $7306, and range of values equal to $43,876–$2304. In four, out of one thousand repetitions, the cost per QALY was positive. Hence, the RHP is robustly dominant against parameter uncertainty. In addition, the maximum positive value of the incremental cost per QALY was $2304, lower than the willingness to pay of the payer located between 1 and 3 times the value of a Peruvian GDP per capita of $6571 [18] (Fig. 3).

The incremental costs had a ranged from $5510 to $234 with a mean of $2374, a standard deviation of $889, and a RSE of 37%. The incremental QALYs showed less variability with a RSE of 13%, a mean of 0.035, a standard deviation of 0.005, and a range from 0.02 to 0.050. The reason is the parameters that concentrated the largest portion of the variability over the ICER—treatment effect of the intervention, cost of the program, and cost of dialysis—have a greater effect on cost than on utilities.

## Discussion

From a lifetime deterministic CEA, we found that the RHP was cost-saving compared to the usual care with an incremental cost per person-year avoided in dialysis of $2173 and per QALY gained of $21,660, comparing the RHP to usual care. In addition, form the results of the PSA we can confidently conclude that the RHP is a dominant strategy in across multiple scenarios and against parameters uncertainty.

There are two main reasons behind the reported negative ICER. First, the difference in QALYs is very small. At a 30-year projection, the RHP generated 0.04 more QALY per person than the usual care, because the speed of disease progression is slow in both alternatives. This difference constitutes the denominator of the ICER and would create a large ratio. Second, the cost of dialysis is enormous compared to the marginal cost of providing the intervention. While treating a patient in the RHP increases the cost for the payer to $486 compared to usual care, the payers save $13,458 every time a patient delays its start on dialysis. Hence, at the end of the projection, although relatively small, the differences in costs and QALYs favor the RHP.

We used utility scores estimated by Go et al. [16] with a sample of Korean CKD patients. We selected this study because of the similarities between samples characteristics—in terms of age and gender composition—the use of standard gamble for preferences elicitation, and the estimation of a score for hemodialysis initiation [16]. For external validity, a meta-analysis that included 190 publications from all over the world and adjusted for utility elicitation method found a CKD utility score of 0.8, and 0.69 for dialysis [20], close to the scores we used. Additionally, our sensitivity analysis included much lower values for both health status, such as those found in studies conducted in the United States using time-trade-off methods: between 0.69 and 0.67 for the CKD stages, and 0.63 and 0.54 for the dialysis patients [21, 22].

From the institutional information provided by the RHP, we estimated an annual cost of hemodialysis treatment per patient of $13,459. We found two studies that reported cost for hemodialysis in Peru. The first one from 2006 estimated an annual cost of $7128 in a Hospital of the Ministry of Health [23], and the second one from 2005 found the cost to be $7536 for a hospital in EsSalud [24]. Assuming an average yearly inflation of 2.5% for the period, both estimates would be around $10,000 after adjusting for inflation. Considering the uncertainty of the parameter, we conducted a threshold analysis to find at what annual cost of hemodialysis the ICER is equal to zero, and therefore the RHP is no longer dominant; we found this value at $9800 per year. Thus, even if the cost of hemodialysis is at previous levels, the intervention is still a dominant alternative.

This study in no without limitations. First, in the last 5 months of the follow up period, the empirical survival curve for mortality showed a steep drop. None of the parametric survival distributions were able to capture this drop, given their tendency to fit a smooth decay [25]. The most likely reasons behind the drop in the survival curve was patients being lost at follow up, rather than a sudden increase in the mortality rate [8]. Considering this, our survival curves were calibrated to the core progression of the disease for both the transition to dialysis and death. In addition, the PSA consider a level of uncertainty in the parameters of the Weibull distribution to better capture the variability of the estimates (see Annex 4, Additional file 1).

Second, the estimations of annual cost per-patient using a weighted average has two sources of uncertainty. First, the distribution among CKD stages that would influence the final cost due to important differences in the costs between CKD stages. Our stages distribution is consistent with the reported at national level, and to those obtained with larger sample sizes [2, 26, 27]. Therefore, there is no evidence to expect a major change in the distribution across stages. The second source is the cost itself. We used an institutional cost report to limit the uncertainty surrounding the costing data. Additionally, our sensitivity analysis included a 15% two-sided variation to assess robustness in our final estimates.

Third, the follow-up period in the epidemiological study, whose results are inputs in this analysis are probably too short to observe significant variations in the mortality rates. It is expected that an increase in the lifespan of adherent patients would lead to higher treatment costs and therefore make the RHP more expensive than the results we presented and change the cost per QALY that we obtained. Given that the RHP is cost-saving compared to the usual care it is not likely that the results would shift in favor of the usual care, but certainly would present a different scenario.

In 2008 there were several Latin American countries with CKD detection programs, such as Brazil, Argentina, Colombia, and Bolivia, among others [9]. However, in our literature review, we could not find any economic evaluations related to them. There is a lack of information assessing the economic impact of prevention strategies in the Region. The ISPOR 6th Latin America Conference in Sao Paulo expressed the necessity to produce more evidence of the economic impact of these strategies with the final aim to better allocate resources [28]. We hope our findings help filling this gap and show the clinical and economic impact of prevention interventions to prevent CKD progression to policymakers. However, additional research would be needed to determine to what extend the results showed here are applicable to other populations; in particular, the average treatment effect and the annual cost of dialysis.

Several calls have been made for strategies that aim to reduce the incidence of patients needing RRT in Latin America, given the historic economic disparities that directly affect the treatment coverage [10, 29]. The RHP is a cost-saving strategy that reduces the risk of disease progression and the need for RRT. The RHP relies on a multidisciplinary team that provides effective management of the CKD and associated comorbidities. Moreover, it promotes coordination between primary and specialized care providers to face the CKD as a public health problem. All of these features are aligned with recommendations for effective secondary care [28, 30].

### Conclusions

The RHP showed to be a dominant alternative against the standard of care and robust to changes in the parameters’ values. As such, is a well-positioned strategy to increase the years lived without requiring dialysis and consequently reducing the average treatment costs in the long-term. Our results should be used to inform the decision-making process of continuing the RHP. Although these results are promising—especially setting facing resources constrained and limited access to RTT—additional research is needed to determine to what extend the RHP could be applied with similar success in other settings.

## Supplementary Information

**Additional file 1: Annex 1.** Empirical and Fitted survival curves for the transition to dialysis in the control group, Hospital E. Rebagliati Network, Lima, Peru, from Jan 2013 to Dec 2017. **Annex 2.** Empirical and Fitted survival curves for the mortality for all causes in the control group, Hospital E. Rebagliati Network, Lima, Peru, from Jan 2013 to Dec 2017. **Annex 3.** Transition to dialysis survival curve: selected fitted curve and confidence intervals. **Annex 4.** Mortality survival curve: selected fitted curve and confidence intervals.

## Data Availability

This is a modeling analysis with all parameters needed to replicate the results presented in the manuscript: “Methods” Section for the disease models, and “Results” Section for the unitary costs and utility scores. No repositories were generated for this study. We are submitting an Additional file document that contains 4 Annex to expand on the results presented in the study.
