# Supplementary material for: Cost-effectiveness analysis of a strategy to delay progression to dialysis and death among chronic kidney disease patients in Lima, Peru
Source: Cost Eff Resour Alloc. 2021 Oct 10;19:70. doi: 10.1186/s12962-021-00317-0 (PMC8504107; doi:10.1186/s12962-021-00317-0)
Supplement: Supplementary file 1 — Additional file 1: Annex 1. Empirical and Fitted survival curves for the transition to dialysis in the control group, Hospital E. Rebagliati Network, Lima, Peru, from Jan 2013 to Dec 2017. Annex 2. Empirical and Fitted survival curves for the mortality for all causes in the control group, Hospital E. Rebagliati Network, Lima, Peru, from Jan 2013 to Dec 2017. Annex 3. Transition to dialysis survival curve: selected fitted curve and confidence intervals. Annex 4. Mortality survival curve: selected fitted curve and confidence intervals. [file 12962_2021_317_MOESM1_ESM.docx]

**Supplemental Material**

Annex 1. Empirical and Fitted survival curves for the transition to dialysis in the control group, Hospital E. Rebagliati Network, Lima, Peru, from Jan 2013 to Dec 2017.

Annex 2. Empirical and Fitted survival curves for the mortality for all causes in the control group, Hospital E. Rebagliati Network, Lima, Peru, from Jan 2013 to Dec 2017.

Annex 3. Transition to dialysis survival curve: selected fitted curve and confidence intervals.

Annex 4. Mortality survival curve: selected fitted curve and confidence intervals.

**Annex 1. Empirical and Fitted survival curves for the transition to dialysis in the control group, Hospital E. Rebagliati Network, Lima, Peru, from Jan 2013 to Dec 2017.**

1. Graphical representation
2. Summary of values

|  | Log logistic | Log lognormal | **Weibull** | Exponential |
| --- | --- | --- | --- | --- |
| AIC | 4059.5 | 4074.9 | **4059.3** | 4061.1 |
| BIC | 4067.2 | 4083.6 | **4066.9** | 4068.7 |
| Lambda | 0.008 | 7.058 | **0.008** | 0.008 |
| Gamma | 0.944 | 2.985 | **0.938** |  |

*AIC: Akaike Information Criteria for goodness of fit; BIC: Bayesian Information Criteria for goodness of fit. We choose the curve with the lowest AIC and BIC.*

*AIC and BIC rounded to the closest first decimal position; Distribution parameters lambda and gamma, rounded to the closest third decimal position.*

**Annex 2. Empirical and Fitted survival curves for the mortality for all causes in the control group, Hospital E. Rebagliati Network, Lima, Peru, from Jan 2013 to Dec 2017.**

1. Graphical representation
2. Summary of values

|  | Log logistic | Log lognormal | **Weibull** | Exponential |
| --- | --- | --- | --- | --- |
| AIC | 17765.2 | 18002.9 | **17730.2** | 17754.4 |
| BIC | 17772.9 | 18010.6 | **17737.9** | 17762.1 |
| Lambda (λ) | 0.050 | 3.302 | **0.045** | 0.050 |
| Gamma (γ) | 1.136 | 2.028 | **1.098** |  |

*AIC: Akaike Information Criteria for goodness of fit; BIC: Bayesian Information Criteria for goodness of fit. We choose the curve with the lowest AIC and BIC.*

*AIC and BIC rounded to the closest first decimal position; Distribution parameters lambda and gamma, rounded to the closest third decimal position.*

**Annex 3. Transition to dialysis survival curve: selected fitted curve and confidence intervals.**

**Annex 4. Mortality survival curve: selected fitted curve and confidence intervals.**
